# Insights into the Interactions among Roots, Rhizosphere, and Rhizobacteria for Improving Plant Growth and Tolerance to Abiotic Stresses: A Review

**DOI:** 10.3390/cells10061551

**Published:** 2021-06-19

**Authors:** Naeem Khan, Shahid Ali, Muhammad Adnan Shahid, Adnan Mustafa, R. Z. Sayyed, José Alfredo Curá

**Affiliations:** 1 Department of Agronomy, Institute of Food and Agricultural Sciences, University of Florida, Gainesville, FL 32611, USA; 2 College of Life Sciences, Northeast Forestry University, Harbin 150040, China; shahidsafi926@gmail.com; 3 Department of Agriculture, Nutrition and Food Systems, University of New Hampshire, Durham, NH 03824, USA; muhammad.shahid@unh.edu; 4 Biology Center CAS, SoWa RI, Na Sadkach 7, 370-05 České Budějovice, Czech Republic; adnanmustafa780@gmail.com; 5 Department of Microbiology, P.S.G.V.P. Mandal’s, Arts, Science, and Commerce College, Shahada 425409, India; sayyedrz@gmail.com; 6 Facultad de Agronomía, Universidad de Buenos Aires, Av. San Martín 4453, Ciudad Autónoma de Buenos Aires C1417DSE, Argentina; acura@agro.uba.ar

**Keywords:** root, rhizosphere, rhizobacteria, root morphology, abiotic stresses

## Abstract

Abiotic stresses, such as drought, salinity, heavy metals, variations in temperature, and ultraviolet (UV) radiation, are antagonistic to plant growth and development, resulting in an overall decrease in plant yield. These stresses have direct effects on the rhizosphere, thus severely affect the root growth, and thereby affecting the overall plant growth, health, and productivity. However, the growth-promoting rhizobacteria that colonize the rhizosphere/endorhizosphere protect the roots from the adverse effects of abiotic stress and facilitate plant growth by various direct and indirect mechanisms. In the rhizosphere, plants are constantly interacting with thousands of these microorganisms, yet it is not very clear when and how these complex root, rhizosphere, and rhizobacteria interactions occur under abiotic stresses. Therefore, the present review attempts to focus on root–rhizosphere and rhizobacterial interactions under stresses, how roots respond to these interactions, and the role of rhizobacteria under these stresses. Further, the review focuses on the underlying mechanisms employed by rhizobacteria for improving root architecture and plant tolerance to abiotic stresses.

## 1. Introduction

Stress is any environmental factor that can adversely affect plant growth and development and decrease the final yield. All the major abiotic stresses lead to the major declines in the yield of globally important crop plants. Drought stress affects leaf expansion, stem elongating, root proliferation, and reduces water and nutrient uptake [1]. Water stress for a prolonged duration also declines leaf water potential and stomatal closing and opening, delays flowering, and decreases seed number and size [2,3,4]. Salt stress is the most stubborn among all abiotic stresses and has prolonged deleterious effects on glycophytes [5,6]. Many plants cannot endure salt concentrations of more than 200 mM [7], as high salinity reduces the rate of seed germination, the establishment of seedlings, vegetative growth, and increases the osmotic pressure, with ion toxicity ultimately leading to oxidative damage [8,9,10]. Heat stress affects overall plant morphology, physiology, and biochemistry, leading to stunted plant growth and a reduction in plant biomass and productivity [11,12]. Similarly, heavy metals have direct and indirect effects on plant growth, significantly reduce plant growth, and disturb various physiological and molecular mechanisms of plants, resulting in chlorosis, senescence, and an inhibition of chlorophyll and photosynthesis [13,14,15]. The above-mentioned stresses are collectively known as abiotic stresses, which are a pretesting threat to crop growth and agriculture and responsible for great crop yield loss and future food safety [16,17,18].

**Figure 1:**
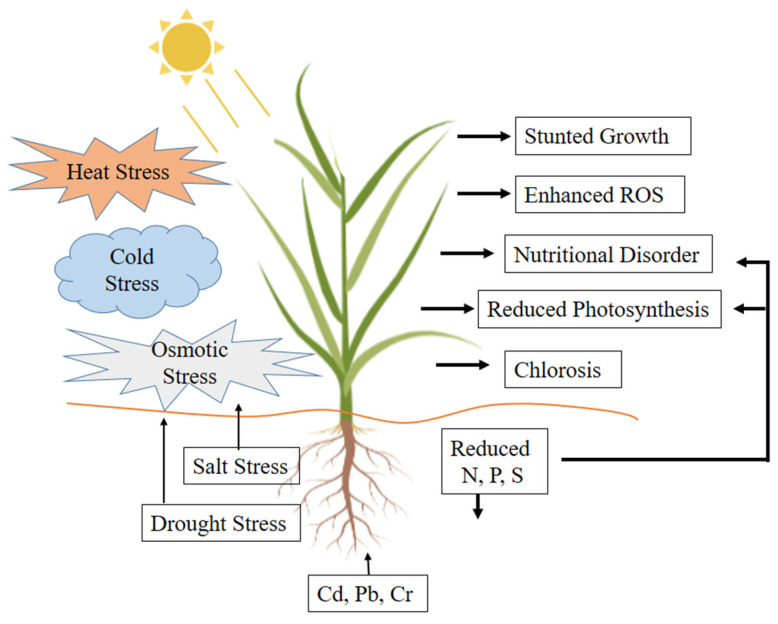
Adverse effects of abiotic stresses on root and shoot growth. Abiotic stresses adversely affect root growth, which results in an overall decrease in plant growth due to an extreme deficiency of water and nutrients in the aboveground parts of the plant.

Plant roots interconnect with a specific group of soil microorganisms that inhabit the root vicinity, known as the rhizosphere. The rhizosphere is considered one of the most composite ecosystems on earth, containing millions of microbial cells—but the number changes according to plant genotype and growth stages [19]. In the rhizosphere, plant roots secrete various compounds that act as a chemical attractant for soil microorganisms [20]. These root exudates also modify the physico-chemical properties of the soil and thus, adjust the structure of soil microbial communities in the close vicinity of the root surface [21,22]. Rhizobacteria that inhabit the rhizosphere alleviate the influences of abiotic stresses on plants through a number of different mechanisms, which include alterations in phytohormone levels, metabolic adjustments, antioxidant defenses, bacterial exopolysaccharides (EPS), and protecting and improving the root growth. These microorganisms can modulate the expression of plant metabolites and improve their photosynthetic, carbohydrate, and protein content, thus improving the yield-related traits under stress [23]. They improve plant growth by enhancing nutrient and water uptake from the soil even under stressful environments [24]. They also improve the phosphate and nitrate reductase activities under water-stressed conditions [25] and limit the Na^+^ accumulation under salt stress [26]. Furthermore, they indirectly promote plant growth by decreasing the damaging effects of pathogenic organisms by producing antagonistic substances (Figure 1) [27]. The PGPR *Burkholderia phytofirmans* improved the photosynthesis and defense responses of *Arabidopsis thaliana* to pathogenic attack [28], whereas Pseudomonas putida improved the systemic resistance and priming of wheat plants to pathogen attack [29].

In this review, we summarize and discuss the current understanding of root–rhizosphere and rhizobacterial interactions to abiotic stresses. We first summarize the impacts of abiotic stresses on overall plant growth and yield. We then elucidate the role of rhizobacteria under abiotic stresses and evaluate the strategies of rhizobacteria for improving root growth and plant tolerance mechanisms.

## 2. The Root–Rhizosphere and Rhizobacterial Alliance

Root-associated rhizobacterial communities play an important role in the maintenance of plant health under abiotic stresses. Plant–microbial associations happen at the rhizosphere. The rhizosphere consists of both beneficial and pathogenic microorganisms. This rhizobacterial community of the rhizosphere changes with changes in soil properties [29]. Interactions between rhizobacteria in the rhizosphere have intuitive effects (i.e., improvement of the soil nutrient content, remediation of HMs, minimization of soil disturbances and root growth and soil immune responses) on soil health and improve the nutritional status of the soil, which is important for better plant growth [30]. A surfeit of these interactions between the roots, rhizosphere, and rhizobial microbes also improves root growth and proliferation, which play a critical role in the exchange of resources between the shoots and the soil environment [31,32]. Rhizobacteria also benefit crop production by reducing the dependency on chemical fertilizers to attain high production yields.

This rich rhizosphere–rhizobacterial interaction defends root exudates, which consist of various organic compounds that attract the microorganisms towards the root vicinity [33,34]. Root exudation arbitrates plant–microbe interactions by root colonization and the promotion of root growth. As Neal et al. [35] reported, there is an increase in the removal of benzoxazinone from the maize rhizosphere due to the presence of *P. putida*. Root exudates contain a wide array of chemical constituents, including amino acids, peptides, sugars, enzymes, vitamins, organic acids, and different types of primary and secondary metabolites [36,37]. The microbial soil diversity depends on the type and composition of root exudate, which supports the growth of useful microorganisms that can assist in plant health and their productivity, while, in other cases, some root exudates also prevent the growth of harmful microbes [38,39,40,41,42]. The proteome data also provide evidence on the biological process that occurs in the rhizosphere, as Baysal et al. [43] carried out a proteomic approach for studying the control of soil-borne pathogens with the help of *Bacillus* species. Bona et al. [44] used the metaproteome approach for studying the microbial communities and their activities in the rhizosphere. They reported the rhizosphere proteome of *Vitis vinifera*, where they found that the bacterial species belonging to the *Bacillus*, *Pseudomonas*, *Bradyrhizobium*, *Streptomyces*, and *Bulkhorderia* genuses are more active in protein expression and their rhizospheres have more metabolic processes and regulation.

The root exudate strigolactone is also an important signaling molecule that regulates primary root and root hair length. They are present in root exudates of monocotyledonous and dicotyledonous plants and are involved in mutualistic interactions with arbuscular mycorrhizal fungi in the rhizosphere [45]. Strigolactones induce hyphal branching near the host plant, which enhances the chances of interactions between the host plant roots and fungi [46]. They may also play an important role in legume–rhizobia symbiosis [47,48].

Among all these root exudates, the most important are organic acids, which not only act as a source of energy for microbial–cellular metabolism but also act as intermediary compounds in bio-geochemical cyclic reactions taking place in the rhizosphere [49,50]. The low-carbon molecules of root exudates act as the originator for the biosynthesis of rhizobacterial-produced phytohormones, whereas tryptophan (Trp) present in the root exudates acts as a precursor for the production of indole-3-acetic acid (IAA), and is mostly present in the root tip region [51]. In addition, the precursor for ethylene, aminocyclopropane-1-carboxylic acid (ACC), also oozes out from plants and can be utilized as a source of nitrogen and carbon by rhizobacteria [52,53,54]. Other compounds identified as flavonoids are released by the roots of leguminous plants, persuading the transcription of rhizobia Nod factors (NF). Nod factors are responsible for the formation of root hairs and also play an important role in nodule initiation [55,56,57,58].

The root–rhizosphere and rhizobacterial interactions also influence plant responses to environmental stresses [59,60,61]. These rhizobacterial species are reported to impart abiotic stress lenience by up or down-regulating the stress-responsive genes, such as S-adenosyl-methionine synthetase, ascorbate peroxidase, and heat shock proteins [62,63,64,65]. The root–rhizobial association also prompts resistance against root herbivores and guards the roots against a number of diseases [66]. The effects of rhizobacteria on the growth of root hairs and root system architecture were inspected on seedlings grown in vitro in upright agar plates comprising a mineral medium inoculated with a 10^8^ cfu per mL of rhizobacteria. The results of the experiment showed significant positive effects of the inoculated rhizobacteria on root hairs and architecture under in vitro conditions [67,68,69,70].

**Figure 2:**
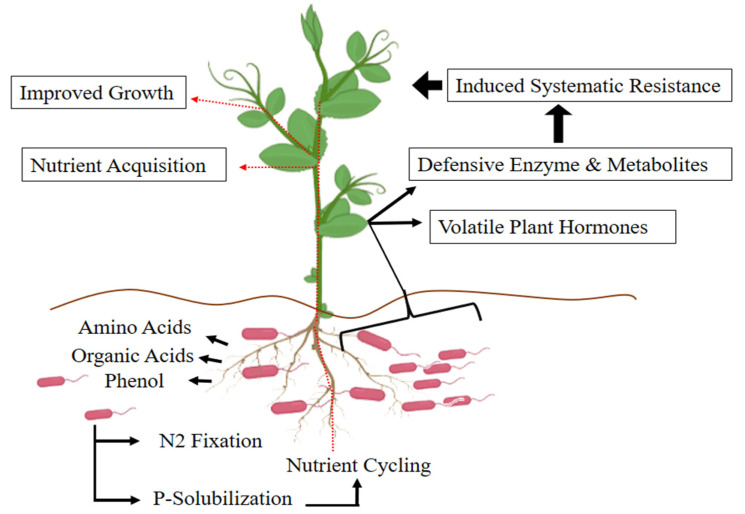
Mechanisms employed by rhizobacteria for increases in plant growth and tolerance to abiotic stresses. Rhizobacteria improve nutrient content and nutrient cycling and help plants to withstand harsh environmental conditions.

The role of root–rhizosphere and rhizobacterial interactions is essential for plant growth promotion, nutrient acquisition, and yield quality [71]. It is apparent that mutual communications occur among plants, soil, and microorganisms, and all such interactions are intricate and should be accounted for useful outcomes in terms of plant growth and soil health (Figure 2) [72,73,74,75,76].

## 3. Effects of Abiotic Stresses on Root Growth and Rhizosphere

Abiotic stresses adversely affect plant growth and development as well as the overall growth and morphology of roots, which not only affects the crop quality but also the final yield. An increase in carbon dioxide (CO_2_) levels results in global climate change, consisting of a rise in temperatures and disturbing weather patterns that significantly affect the plant rhizosphere [77,78,79,80].

Plant roots are the major organs responsible for nutrient and water acquisition and maintaining normal plant growth and yield [81,82]. However, abiotic stresses result in poor root growth, which results in decreased water and nutrient uptake. Drought stress has more severe effects on plant roots than any other stress and significantly reduces its biomass [83,84]. Salinity causes ion toxicity due to an excess of Na^+^ and Cl^−^, which also damages root growth and development [85,86]. High temperatures adversely affect the root architecture and the roots’ interactions with the surrounding microorganisms (Figure 1), whereas a decrease in temperature at the root zone adversely affects the process of nodulation and N-fixation [87,88]. Similarly, the presence of heavy metals (HMs) in the rhizosphere has toxic effects on root growth (Figure 1) [89]. Among the HMs, lead (Pb) is the most widespread, causing inhibition of cell division in the root tip and rapid inhibition of root growth [90]. The presence of a high concentration of cadmium (Cd) in the rhizosphere causes visible injuries to the root and shoot, browning of the root tips, and chlorosis in plant shoots [91,92,93]. Similarly, chromium (Cr) toxicity also causes chlorosis in newly developing leaves, and injury to roots [94,95].

### Role of Rhizobacteria under Abiotic Stresses

Rhizosphere microorganisms, mainly beneficial bacteria, can increase plant performance under stressful conditions and, consequently, improve soil health and enhance root growth and plant yield [96]. Rhizobacteria either exert a direct stimulation on root and overall plant growth by fixing nitrogen, the production of plant hormones, and sequestering iron and solubilizing phosphate [97,98,99]. The microbial-produced phytohormones promote root growth and alter root architecture, triggering an increase in root surface area [100]. This is considered one of the basic mechanisms employed by root-associated bacteria for the increases in nutrient uptake. In the rhizosphere, plant–rhizobacteria interactions assist plants through the induction of systemic resistance against pathogens and 1-aminocyclopropane 1-carboxylic acid (ACC)-deaminase activity. Such stimuli of rhizobacteria can benefit plant defense against pathogens and can also improve overall plant yield (Figure 2; Table 1) [101,102,103,104,105].

It has also been reported by Marulanda et al. [125] that rhizobacterial-inoculated plants exhibit significant increases in plant growth and yield, as well as in drought tolerance to semi-arid and arid environments. The application of *Phyllobacterium brassicacearum* strain STM196 to *Arabidopsis thaliana* improved its resistance to moderate water deficit stress by modulating the rate of transpiration and delaying maturity [126]. The inoculation of plants with rhizobacteria helps in the mitigation of the deleterious effects of various stresses by assisting them in the acquisition of less available nutrients and by increasing the levels of plant growth regulators [12]. Microorganisms with the capability to persist under severe environmental conditions are more active at vindicating the undesirable impacts of drought on crop plants [127]. Niu et al. [128] reported that drought-tolerant strains are capable of producing exopolysaccharides (EPS), which stimulate seed germination and seedling growth under drought stress. Among all PGPR strains, *Pseudomonas fluorescens* has the highest capability of producing EPS and ACC deaminase. Recently, Batool et al. [129] reported the effects of rhizobacteria in reducing the impacts of drought and maintaining the higher growth and physio-chemical properties of the plants. They noted a higher growth rate and leaf area and an increase in dry matter production in inoculated plants. The isolated PGPR-HAS31 maintained higher chlorophyll, photosynthesis, soluble proteins, sugars, and enzymatic activities in relation to uninoculated plants.

Kumar et al. [130] studied the effects of salt-tolerant (ST) bacterial strains. Their results exposed the inoculation of paddy plants with the rhizobacteria *Pseudomonas aeruginosa*, and *Lysinibacillus* sp. boosted the seedlings’ growth under salinity stress. *Pseudomonas aeruginosa* exhibited more profound effects than other species and significantly improved the rate of seed germination and the lengths of shoots and roots. Shultana et al. [131] measured the effects of rhizobacterial strains isolated from the saline rice field in Malaysia on the growth and yield of rice plants. Their results revealed significant useful effects of bacterial inoculation on the rate of transpiration, photosynthesis, and stomatal conductance, which also resulted in a higher increase in yield. The most frequently used halotolerant rhizobacteria are *Azotobacter*, *Acinetobacter*, Bacillus sp., *Pseudomonas* sp., *Rhizobium* sp., and *Serratia* sp., which employ different mechanisms, including N-fixation, P-solubilization, and siderophore formation [132,133,134,135]. Similarly, many different reports revealed that halotolerant microbes significantly enhanced the growth of many crops under both normal and saline conditions [136,137]. Various ST rhizobacterial species improve the salt tolerance in plants by the production of different types of osmolytes and antioxidant enzymes and synthesizing ACC deaminase [138,139,140].

Temperature is another important variable that influences plant root growth. Fluctuations in the temperature of the root zone also alter shoot growth responses by inducing changes in the temperature of the shoot apical meristems [77]. It has adverse effects on the plasma membrane, photosynthesis, phytohormones, and enzyme activity. However, the rhizosphere microbes have the ability to mitigate the adverse effects of high temperature stress. They protect their membranes and nucleic acids under such conditions and contribute to normal plant growth. Some microorganisms are better in the production of biofilm and can help plants to tolerate high salt and temperature stress [141]. Moreover, *B. subtilis* Co1-6 and *P. polymyxa* Mc5Re-14 showed better production of the bioactive secondary metabolite apigenin-7-*O*-glucoside, whereas inoculation with *Pseudomonas* sp. strain AKM-P6 and *P. putida* strain AKM-P7 enhanced the tolerance of sorghum and wheat seedlings to high temperature stress due to the synthesis of high-molecular-weight proteins, and also improved the levels of cellular metabolites [142,143]. Zhu et al. [144] observed positive physiological effects of the arbuscular mycorrhizal fungus *Glomus etunicatum* on *Zea mays* plants at a range of different temperatures (5–40 °C) when compared with uninoculated plants. Similarly, Pedranzani et al. [145] showed an increase in antioxidants in the shoots and roots of *Digitaria eriantha* and a reduction in cellular lipid peroxidation in plants inoculated with the arbuscular mycorrhizal fungus *Rhizophagus irregularis* under cold stress (4 °C).

Soil microbes maintain an efficient flow of water and nutrients to plants under heat stress conditions [146], whereas thermotolerant phosphate solubilizing bacteria act as biofertilizers in agriculture and are involved in the biogeochemical cycling of phosphorus [147]. One of the common mechanisms inked by rhizosphere microorganisms for reducing the effects of heat stress in plants is the induction of osmoprotectants and heat shock proteins (HSP). The modulation of the levels of phytohormones, secondary metabolites, and the production of ROS are some of the important mechanisms employed by rhizosphere microorganisms for combating the adverse effects of heat stress. Kang et al. [148] reported an increase in the content of GA and ABA and the reduction in the content of jasmonate and salicylate in pepper plants inoculated with a gibberellin-producing PGPR. This alteration in the content of phytohormone/plant growth regulators resulted in an increase in plant growth under low temperature stress conditions. Issa et al. [149] reported that bacterium *Burkholderia phytofirmans* significantly enhanced the production of phenolics, proline, and starch under heat stress and was able to protect the tissues of tomato against heat. Rodriguiz et al. [150] also reported *Curvularia protuberate*-induced heat stress tolerance in tomatoes. Gram-positive microorganisms possess heat-resistant spores that are used in the formulation of stable and dry powder products [151].

Rhizobacteria are known to affect the movement and accessibility of HMs by releasing various chelating agents or by the process of acidification, phosphate solubilization, and redox reaction and thus, enhance the phytoremediation of HMs [152]. The aptitude of microorganisms to degrade pollutants largely depends on the pH, temperature, and moisture content of the environment in which they live [153]. Microorganisms can also cleanse metals by valence conversion, volatilization, or extracellular chemical precipitation [40]. However, the co-inoculation of *Bacillus subtilis* was found to be more effective against the remediation of HMs than a single inoculation. Some bacterial species produce iron-chelating substances called siderophores, which enhance the mobility and reduce the bioavailability of metals [154]. Sulfate-reducing bacteria have the ability to convert sulfate to hydrogen sulfate, which then reacts with heavy metals and converts them to insoluble forms of metal sulfides [155]. The oxalate crystals produced by mycorrhizal fungi are also known to immobilize and detoxify HM [156].

Tiwari et al. [157] reported that plant-associated bacteria reduce the accumulation of various metals in plant tissues and also assist in reducing metal availability in the soil through a number of different mechanisms. The practice of rhizobacteria in combination with plants is estimated to deliver high efficacy for phytoremediation [158,159,160]. Khanna et al. [161] also reported the role of *Pseudomonas aeruginosa* and *Burkholderia gladioli* in the alleviation of Cd stress (0.4 mM) in the 10-day old seedlings of *L. esculentum*. They revealed the adverse effects of Cd stress on root and shoot growth and on plant biomass. However, the bacterial-inoculated plants showed improved plant growth and resistance to Cd toxicity. The usage of these beneficial rhizobacteria is reflected as one of the most capable approaches for harmless crop management practices in HM-contaminated soils. Plant–microbe interactions help in adapting plants to metalliferous environments and increase microbial-assisted metal tolerance.

## 4. Strategies of Rhizobacteria for Improving Root Architecture under Stresses

Rhizobacteria efficiently colonize the roots of crop plants and enhance their growth by a number of different direct and indirect mechanisms. The alteration of root system architecture by root-associated rhizobacteria involves the assembly of phytohormones, for example, auxin, gibberellins, and other signaling molecules that lead to greater lateral root branching and growth of root hairs. As these rhizobacteria attach to the plant root surface, they convert root exudates into phytohormones [162,163,164,165,166]. The composition of the root exudates change along with the plant development; hence, the rhizo-microbiome alignment varies consequently [167,168,169]. They also show the antagonistic activities against the phytopathogenic microorganisms by producing siderophores, enzymes, the synthesis of antibiotics, antifungal compounds, and essential nutrients, thus improving the root architecture under all these stressful conditions [170,171,172].

**Figure 3:**
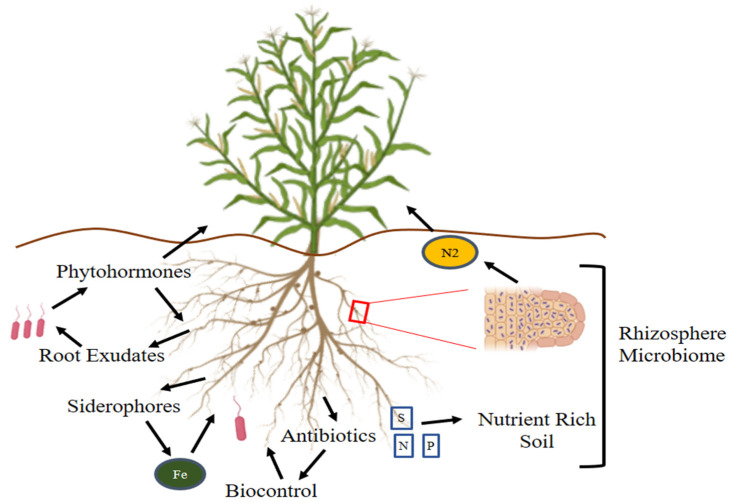
Strategies of PGPR for improving root architecture and overall plant growth under abiotic stresses. These microorganisms form a rhizosheath around the roots and produce antibiotics and biocontrol agents, thus protecting the roots from the adverse effects of environmental stresses.

The root colonization pattern of rhizobacteria like *Bacillus* and *Pseudomonas* has been well-described in numerous plants, including tomato [173], cucumber [174], *Arabidopsis thaliana* [175], wheat [176], and grape [177]. Erturk et al. [178] studied the effects of various strains of *Bacillus* on rooting and root growth in kiwifruit. The highest rooting ratios (47.5%) were obtained as a result of Bacillus RC03 and *Bacillus simplex* RC19 treatments. The inoculation of wheat and maize plants with these bacterial species also delayed the onset of the drought symptoms on plants. Both of the applied rhizobacterial species were synergistic to root branching and length, compared to the control. *Enterobacter* sp. demonstrated greater effects on root branching, length, and diameter when compared to the control (Figure 3) [179,180,181,182].

## 5. Stress Responsive Metabolites Mediated by Rhizobacteria

Plants experience diverse abiotic stresses throughout their life cycle that need to be handled in order to survive. Abiotic stress lenience has been studied in relation to rhizobacteria in order to offer a biological understanding of the alteration and persistence of rhizobacteria under such stresses [183,184]. Several stress tolerance mechanisms have been considered for rhizobacterial-arbitrated stress tolerance in plants. It has been reported that rhizobacterial inoculation alters the metabolic expression in plants under stress and helps in activating stress-responsive genes and metabolites [185]. The potential of rhizobacteria producing stress-related metabolites is gaining importance these days. They also have the ability to modulate the transcriptional machinery responsible for stress tolerance in plants. Their involvement in the upregulation of ABA-signaling cascades that lead to the expression of *TaWRKY* and *TaMYB* has been reported previously [68,186]. Many genetic studies have been carried out in plants grown under abiotic stresses to characterize the bacterial-mediated changes in plants at genetic and metabolic levels [187,188]. Previously, the genetic studies of drought stress tolerance were categorized by means of molecular and genetic approaches in pepper plants [184,189,190,191,192,193,194].

**Figure 4:**
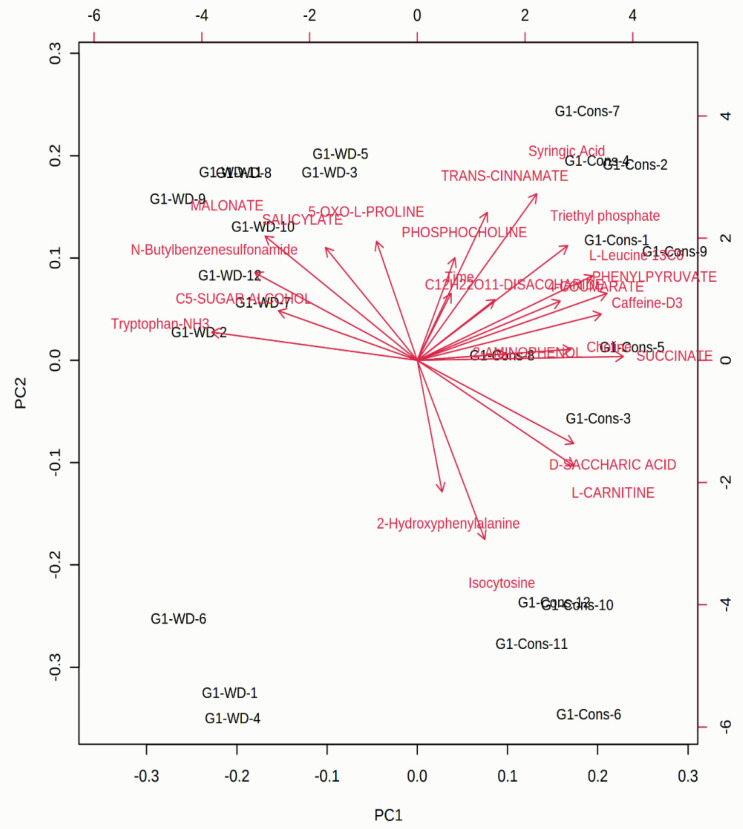
A PCA-based biplot showing the associations among different metabolites induced by plant growth-promoting rhizobacteria (PGPR) in chickpea leaves grown under stress conditions. The figure was generated by uploading data files to the MetaboAnalyst 3.0 server (http://www.metaboanalyst.ca accessed on 10 February 2021). We selected normalization by sum, log transformation, and auto-scaling for the analysis.

A large number of secondary metabolites, such as compatible solutes and volatile organic compounds (VOCs), have been reported to be from halotolerant rhizobacteria that are crucial for bettering the adverse effects of salinity stress in plants and their associated partners [195]. Halotolerant rhizobacteria employ key mechanisms for stress tolerance, which include osmotic adjustments at a cellular level, regulation in ionic transportation, and maintaining homeostasis by reducing the toxic effects of sodium (Na^+^) and chlorine (Cl^–^) ions [196]. Moreover, these microbes synthesize different types of volatile compounds and antifungal or antibacterial metabolites, for example, sugar, betaines, amino acids, and polyols, which help plants to withstand harsh environmental conditions [197,198,199,200]. Some of the halotolerant bacteria can endure stress caused by high salinity due to their innate ability to accrue some of the vital compatible osmolytes essential for retaining intracellular osmotic homeostasis that benefit them to persist under high salinity, and they are also liable for supporting plant growth and survival under such stresses (Figure 4) [201,202,203,204,205].

The overproduction of reactive oxygen species (ROS) under stress conditions alters redox states, causes damage to DNA, proteins, and membrane fluidity, and lastly, causes cell death [189]. Various growth-promoting rhizobacterial species are described to endure oxidative stress with the support of antioxidant enzyme activity. Sandhya et al. [206] reported an increase in the activity of ascorbate peroxidases (APX) in *Enterobacter* inoculated tomato seedlings grown under high saline conditions. Higher catalase (CAT) and superoxide dismutase activities were recorded in bacterial inoculated gladiolus plants when compared to the control [207,208].

Endophytic bacteria are capable of synthesizing nitrogenase enzymes under HM stress and destitute nitrogen conditions by giving ample nitrogen to connected plants. Doty et al. [209] also isolated endophytic genera (*Acinetobacter*, *Burkholderia*, *Rahnella*, and *Sphingomonas)* from *Populus trichocarpa* and *Salix sitchensis*, with the ability to synthesize nitrogenase enzymes, and were capable to fix atmospheric nitrogen [210]. The production of citric acid, gluconic acid, and oxalic acid by rhizobacteria plays an effective role in the mobilization of heavy metals, thus protecting the plant roots from the lethal effects of HMs [211,212,213]. Paredes-Páliz et al. [214] selected biofilm-forming rhizobacteria based on their ability for metal tolerance and applied them to *Spartina densiflora*. The inoculated plants were then allowed to grow for four months and then harvested. The frozen harvested plant parts were used for the determination of enzyme assays and gene expression. They noted increases in the activity of SOD, CAT, and APOX and a 2-fold increase of thiobarbituric acid reactive substances (TBARs) that resulted in membrane and cell damage. However, the created oxidative stress index (OSI) was significantly decreased (>50%) upon inoculation.

Changes in gene expression in relation to ethylene biosynthesis have been reported in rhizobacterial-inoculated plants grown under abiotic stresses [215,216,217,218]. The existence of ethylene (ET) is vital for normal plant growth and fruit ripening, but under stress conditions, the production of ethylene significantly increases, which has negative effects on seed germination and root growth [219,220]. However, ACC deaminase-containing rhizobacteria can hydrolyze ACC, the precursor of ET, thus decreasing the extra ethylene production under stress and saving plants from its inhibitory effects [221,222,223]. Beneficial rhizobacteria enhance the synthesis of proline in abiotically stressed plants. The most important proline synthesizing rhizobacteria are *Burkholderia* [224], *Arthrobacter*, and *Bacillus* [225].

## 6. Conclusions

Soil microbiomes and especially rhizobacteria possess different mechanisms by which they improve soil health, root growth, and the tolerance of plants to various abiotic stresses. The ability of these bacteria to survive under abiotic stresses makes them a brilliant candidate for sustainable agriculture. They improve root access to nutrients and water and improve their translocation to the above-ground parts of the plants, leading to overall improvements in plant growth and yield. These bacterial strains mitigate the adverse effects of abiotic stress by producing different types of metabolites, including phytohormone, exopolysaccharides, siderophores, antioxidant enzymes, and volatile compounds. Improvements in plant tolerance to abiotic stresses will result in increased yields and production of crops, even under stressful environments. This can be achieved via the search, selection, and engineering of rhizobacterial species capable of resistance to abiotic stresses.

## Figures and Tables

**Table 1:** List of rhizobacteria species responsible for abiotic stress tolerance in common crop plants.

Crop	Stress	Rhizobacteria	References
*Helianthus annuus*	Drought	*Achromobacter xylosoxidans* (SF2) *Bacillus pumilus* (SF3 and SF4)	Castillo et al. [106]
*Oryza sativa*	Drought	*Azospirillum brasilense* Az.39	Ruíz-Sanches et al. [107]
*Vigna radiata*	Drought	*Pseudomonas fluorescens* strain Pf1 *Bacillus subtilis* EPB5, EPB22 and EPB31	Saravanakumar et al. [108]
*Cucurbita pepo*	Drought	*Bacillus circulans ML2, Bacillus megaterium* ML3	El-Meihy [109]
*Zea mays*	Drought	*Klebsiella variicola* F2, *Pseudomonas fluorescens* YX2 *Raoultella planticola* YL2	Gou et al. [110]
*Arachis hypogea*	Salinity	*B. licheniformis* K11	Lim et al. [111]
*Phaseolus vulgaris*	Salinity	*Aneurinibacillus aneurinilyticus, Paenibacillus sp.*	Gupta and Pandey [112]
*Steva rebaundia*	Salinity	*Steptomyces spp.*	Tolba et al. [113]
*Abelmoschus esculentus*	Salinity	*Enterobacter sp.*	Habib et al. [114]
*Lycopersicon esculentum*	Heavy metal	*Pseudomonas aeruginosa, Burkholderia gladioli*	Khana et al. [115]
*Triticum aestivum*	Heavy metal	*Bacillus siamensis*	Awan et al. [116]
*Brassica nigra*	Heavy metal	*Bacillus cereus*	Akhtar et al. [117]
*Pisum sativum*	Heavy metal	*V. paradoxus* 5C-2	Belimov et al. [118]
*Solanum nigrum*	Heavy metal	*Bacillus* genus	He et al. [119]
*Mentha piperita*	Heavy metal	*Alcalegenes faecalis, B. amyloliquefaciens*	Zafar-ul-Haye et al. [120]
*Triticum aestivum*	Heat	*Pseudomonas brassicacearum, Bacillus thuringiensis, Bacillus subtilis*	Ashraf et al. [121]
*Triticum aestivum*	Heat	*Bacillus velezensis* 5113	Abde El-Daim [122]
*Lycopersicon esculentum*	*Heat*	*Bacillus cereus*	Khan et al. [123]
*Solanum tuberosum*	*Salt/Drought/HMs*	*Bacillus pumilus DH 11, Bacillus firmus 40*	Gururani et al. [124]

## Data Availability

All the data generated or analyzed during this study are included in this article.

## References

[1] Farooq M., Wahid A., Kobayashi N., Fujita D.B.S.M.A., Basra S.M.A. (2009). Plant drought stress: Effects, mechanisms and management. Sustainable Agriculture.

[2] Meena K.K., Sorty A.M., Bitla U.M., Choudhary K., Gupta P., Pareek A., Singh D.P., Prabha R., Sahu P.K., Gupta V.K. (2017). Abiotic stress responses and microbe-mediated mitigation in plants: The omics strategies. Front. Plant Sci..

[3] Li G., Zhao H., Liu Z., Wang H., Xu B., Guo X. (2018). The wisdom of honeybee defenses against environmental stresses. Front. Microbiol..

[4] Xu K., Lee Y.S., Li J., Li C. (2019). Resistance mechanisms and reprogramming of microorganisms for efficient biorefinery under multiple environmental stresses. Synth. Syst. Biotechnol..

[5] Negrão S., Schmöckel S.M., Tester M. (2017). Evaluating physiological responses of plants to salinity stress. Ann. Bot..

[6] Egamberdieva D., Lugtenberg B. (2014). Use of plant growth-promoting rhizobacteria to alleviate salinity stress in plants. Use of Microbes for the Alleviation of Soil Stresses.

[7] Timmusk S., Timmusk K., Behers L. (2013). Rhizobacterial plant drought stress tolerance enhancement: Towards sustainable water resource management and food security. J. Food Secur..

[8] Kaushal M., Wani S.P. (2016). Rhizobacterial-plant interactions: Strategies ensuring plant growth promotion under drought and salinity stress. Agric. Ecosyst. Environ..

[9] Kumari B., Mallick M.A., Solanki M.K., Solanki A.C., Hora A., Guo W. (2019). Plant growth promoting rhizobacteria (PGPR): Modern prospects for sustainable agriculture. Plant Health under Biotic Stress.

[10] Subiramani S., Ramalingam S., Muthu T., Nile S.H., Venkidasamy B. (2020). Development of abiotic stress tolerance in crops by plant growth-promoting rhizobacteria (PGPR). Phyto-Microbiome in Stress Regulation.

[11] Barnawal D., Bharti N., Pandey S.S., Pandey A., Chanotiya C.S., Kalra A. (2017). Plant growth-promoting rhizobacteria enhance wheat salt and drought stress tolerance by altering endogenous phytohormone levels and TaCTR1/TaDREB2 expression. Physiol. Plant..

[12] Morcillo R.J., Manzanera M. (2021). The Effects of Plant-Associated Bacterial Exopolysaccharides on Plant Abiotic Stress Tolerance. Metabolites.

[13] Van Loon L.C. (2007). Plant responses to plant growth-promoting rhizobacteria. New Perspectives and Approaches in Plant Growth-Promoting Rhizobacteria Research.

[14] Bhat M.A., Kumar V., Bhat M.A., Wani I.A., Dar F.L., Farooq I., Bhatti F., Koser R., Rahman S., Jan A.T. (2020). Mechanistic insights of the interaction of plant growth-promoting rhizobacteria (PGPR) with plant roots toward enhancing plant productivity by alleviating salinity stress. Front. Microbiol..

[15] Goswami D., Thakker J.N., Dhandhukia P.C. (2016). Portraying mechanics of plant growth promoting rhizobacteria (PGPR): A review. Cogent Food Agric..

[16] Bhattacharyya P.N., Jha D.K. (2012). Plant growth-promoting rhizobacteria (PGPR): Emergence in agriculture. World J. Microbiol. Biotechnol..

[17] Zhu J.K. (2016). Abiotic stress signaling and responses in plants. Cell.

[18] Kosová K., Vítámvás P., Urban M.O., Prášil I.T., Renaut J. (2018). Plant abiotic stress proteomics: The major factors determining alterations in cellular proteome. Front. Plant Sci..

[19] Singhal P., Jan A.T., Azam M., Haq Q.M.R. (2016). Plant abiotic stress: A prospective strategy of exploiting promoters as alternative to overcome the escalating burden. Front. Life Sci..

[20] Pandey P., Irulappan V., Bagavathiannan M.V., Senthil-Kumar M. (2017). Impact of combined abiotic and biotic stresses on plant growth and avenues for crop improvement by exploiting physio-morphological traits. Front. Plant Sci..

[21] Bechtold U., Field B. (2018). Molecular Mechanisms Controlling Plant Growth during Abiotic Stress. J. Exp. Bot..

[22] Yang J., Kloepper J.W., Ryu C.M. (2009). Rhizosphere bacteria help plants tolerate abiotic stress. Trends Plant Sci..

[23] Ismail M.A., Amin M.A., Eid A.M., Hassan S.E.D., Mahgoub H.A., Lashin I., Abdelwahab A.T., Azab E., Gobouri A.A., Elkelish A. (2021). Comparative Study between Exogenously Applied Plant Growth Hormones versus Metabolites of Microbial Endophytes as Plant Growth-Promoting for *Phaseolus vulgaris* L.. Cells.

[24] Ahkami A.H., White R.A., Handakumbura P.P., Jansson C. (2017). Rhizosphere engineering: Enhancing sustainable plant ecosystem productivity. Rhizosphere.

[25] Kohler J., Hernández J.A., Caravaca F., Roldán A. (2008). Plant-growth-promoting rhizobacteria and arbuscular mycorrhizal fungi modify alleviation biochemical mechanisms in water-stressed plants. Funct. Plant Biol..

[26] Wang Q., Dodd I.C., Belimov A.A., Jiang F. (2016). Rhizosphere bacteria containing 1-aminocyclopropane-1-carboxylate deaminase increase growth and photosynthesis of pea plants under salt stress by limiting Na+ accumulation. Funct. Plant Biol..

[27] Sivasakthi S., Usharani G., Saranraj P. (2014). Biocontrol potentiality of plant growth promoting bacteria (PGPR)-Pseudomonas fluorescens and Bacillus subtilis: A review. Afr. J. Agric. Res..

[28] Su F., Villaume S., Rabenoelina F., Crouzet J., Clément C., Vaillant-Gaveau N., Dhondt-Cordelier S. (2017). Different *Arabidopsis thaliana* photosynthetic and defense responses to hemibiotrophic pathogen induced by local or distal inoculation of Burkholderia phytofirmans. Photosynth. Res..

[29] Pérez-de-Luque A., Tille S., Johnson I., Pascual-Pardo D., Ton J., Cameron D.D. (2017). The interactive effects of arbuscular mycorrhiza and plant growth-promoting rhizobacteria synergistically enhance host plant defences against pathogens. Sci. Rep..

[30] Badri D.V., Weir T.L., Van der Lelie D., Vivanco J.M. (2009). Rhizosphere chemical dialogues: Plant–microbe interactions. Curr. Opin. Biotechnol..

[31] Zhang R., Vivanco J.M., Shen Q. (2017). The unseen rhizosphere root–soil–microbe interactions for crop production. Curr. Opin. Microbiol..

[32] Traxler M.F., Kolter R. (2015). Natural products in soil microbe interactions and evolution. Nat. Prod. Rep..

[33] el Zahar Haichar F., Santaella C., Heulin T., Achouak W. (2014). Root exudates mediated interactions belowground. Soil Biol. Biochem..

[34] Semchenko M., Saar S., Lepik A. (2014). Plant root exudates mediate neighbour recognition and trigger complex behavioural changes. New Phytol..

[35] Neal A.L., Ahmad S., Gordon-Weeks R., Ton J. (2012). Benzoxazinoids in root exudates of maize attract Pseudomonas putida to the rhizosphere. PLoS ONE.

[36] Basiliko N., Stewart H., Roulet N.T., Moore T.R. (2012). Do root exudates enhance peat decomposition?. Geomicrobiol. J..

[37] Korenblum E., Dong Y., Szymanski J., Panda S., Jozwiak A., Massalha H., Meir S., Rogachev I., Aharoni A. (2020). Rhizosphere microbiome mediates systemic root metabolite exudation by root-to-root signaling. Proc. Natl. Acad. Sci. USA.

[38] Berlanas C., Berbegal M., Elena G., Laidani M., Cibriain J.F., Sagües A., Gramaje D. (2019). The fungal and bacterial rhizosphere microbiome associated with grapevine rootstock genotypes in mature and young vineyards. Front. Microbiol..

[39] Raklami A., Bechtaoui N., Tahiri A.I., Anli M., Meddich A., Oufdou K. (2019). Use of rhizobacteria and mycorrhizae consortium in the open field as a strategy for improving crop nutrition, productivity and soil fertility. Front. Microbiol..

[40] Dilnashin H., Birla H., Hoat T.X., Singh H.B., Singh S.P., Keswani C. (2020). Applications of agriculturally important microorganisms for sustainable crop production. Molecular Aspects of Plant Beneficial Microbes in Agriculture.

[41] Akiyama K., Hayashi H. (2006). Strigolactones: Chemical signals for fungal symbionts and parasitic weeds in plant roots. Ann. Bot..

[42] Ahemad M., Kibret M. (2014). Mechanisms and applications of plant growth promoting rhizobacteria: Current perspective. J. King Saud Univ. Sci..

[43] Baysal Ö., Lai D., Xu H.H., Siragusa M., Çalışkan M., Carimi F., Da Silva J.A.T., Tör M. (2013). A proteomic approach provides new insights into the control of soil-borne plant pathogens by Bacillus species. PLoS ONE.

[44] Bona E., Massa N., Novello G., Boatti L., Cesaro P., Todeschini V., Magnelli V., Manfredi M., Marengo E., Mignone F. (2019). Metaproteomic characterization of the *Vitis vinifera* rhizosphere. FEMS Microbiol. Ecol..

[45] Breuillin F., Schramm J., Hajirezaei M., Ahkami A., Favre P., Druege U., Hause B., Bucher M., Kretzschmar T., Bossolini E. (2010). Phosphate systemically inhibits development of arbuscular mycorrhiza in *Petunia hybrida* and represses genes involved in mycorrhizal functioning. Plant J..

[46] De Cuyper C., Fromentin J., Yocgo R.E., De Keyser A., Guillotin B., Kunert K., Boyer F.D., Goormachtig S. (2015). From lateral root density to nodule number, the strigolactone analogue GR24 shapes the root architecture of *Medicago truncatula*. J. Exp. Bot..

[47] Peláez-Vico M.A., Bernabéu-Roda L., Kohlen W., Soto M.J., López-Ráez J.A. (2016). Strigolactones in the Rhizobium-legume symbiosis: Stimulatory effect on bacterial surface motility and down-regulation of their levels in nodulated plants. Plant Sci..

[48] Yang J.L., Fan W., Zheng S.J. (2019). Mechanisms and regulation of aluminum-induced secretion of organic acid anions from plant roots. J. Zhejiang Univ. Sci. B.

[49] Yang L.T., Qi Y.P., Jiang H.X., Chen L.S. (2013). Roles of organic acid anion secretion in aluminium tolerance of higher plants. BioMed Res. Int..

[50] Wu D., Zhao M., Shen S., Fu Y., Sasaki T., Yamamoto Y., Wei W., Shen H. (2013). Al-induced secretion of organic acid, gene expression and root elongation in soybean roots. Acta Physiol. Plant..

[51] Li G.X., Wu X.Q., Ye J.R., Yang H.C. (2018). Characteristics of Organic Acid Secretion Associated with the Interaction between Burkholderia multivorans WS-FJ9 and Poplar Root System. BioMed. Res. Int..

[52] Xiang G., Ma W., Gao S., Jin Z., Yue Q., Yao Y. (2019). Transcriptomic and phosphoproteomic profiling and metabolite analyses reveal the mechanism of NaHCO 3-induced organic acid secretion in grapevine roots. BMC Plant Biol..

[53] Pini F., East A.K., Appia-Ayme C., Tomek J., Karunakaran R., Mendoza-Suárez M., Edwards A., Terpolilli J.J., Roworth J., Downie J.A. (2017). Bacterial biosensors for in vivo spatiotemporal mapping of root secretion. Plant Physiol..

[54] Ziegler J., Schmidt S., Chutia R., Müller J., Böttcher C., Strehmel N., Scheel D., Abel S. (2016). Non-targeted profiling of semi-polar metabolites in Arabidopsis root exudates uncovers a role for coumarin secretion and lignification during the local response to phosphate limitation. J. Exp. Bot..

[55] Sugiyama A. (2019). The soybean rhizosphere: Metabolites, microbes, and beyond—A review. J. Adv. Res..

[56] Clemens S., Weber M. (2016). The essential role of coumarin secretion for Fe acquisition from alkaline soil. Plant Signal. Behav..

[57] Chen Y.T., Wang Y., Yeh K.C. (2017). Role of root exudates in metal acquisition and tolerance. Curr. Opin. Plant Biol..

[58] Pii Y., Mimmo T., Tomasi N., Terzano R., Cesco S., Crecchio C. (2015). Microbial interactions in the rhizosphere: Beneficial influences of plant growth-promoting rhizobacteria on nutrient acquisition process. A review. Biol. Fertil. Soils.

[59] Nadeem S.M., Ahmad M., Zahir Z.A., Javaid A., Ashraf M. (2014). The role of mycorrhizae and plant growth promoting rhizobacteria (PGPR) in improving crop productivity under stressful environments. Biotechnol. Adv..

[60] Khan N., Ali S., Zandi P., Mehmood A., Ullah S., Ikram M., ISMAIL M.A.S., BABAR M. (2020). Role of sugars, amino acids and organic acids in improving plant abiotic stress tolerance. Pak. J. Bot..

[61] Chaparro J.M., Sheflin A.M., Manter D.K., Vivanco J.M. (2012). Manipulating the soil microbiome to increase soil health and plant fertility. Biol. Fertil. Soils.

[62] Hashem A., Tabassum B., Abd_Allah E.F. (2019). Bacillus subtilis: A plant-growth promoting rhizobacterium that also impacts biotic stress. Saudi J. Biol. Sci..

[63] Bharti N., Pandey S.S., Barnawal D., Patel V.K., Kalra A. (2016). Plant growth promoting rhizobacteria Dietzia natronolimnaea modulates the expression of stress responsive genes providing protection of wheat from salinity stress. Sci. Rep..

[64] Jatan R., Chauhan P.S., Lata C. (2019). Pseudomonas putida modulates the expression of miRNAs and their target genes in response to drought and salt stresses in chickpea (*Cicer arietinum* L.). Genomics.

[65] Gontia-Mishra I., Sapre S., Sharma A., Tiwari S. (2016). Amelioration of drought tolerance in wheat by the interaction of plant growth-promoting rhizobacteria. Plant Biol..

[66] Maheshwari D.K., Dheeman S., Agarwal M. (2015). Phytohormone-producing PGPR for sustainable agriculture. Bacterial Metabolites in Sustainable Agroecosystem.

[67] Prieto P., Schilirò E., Maldonado-González M.M., Valderrama R., Barroso-Albarracín J.B., Mercado-Blanco J. (2011). Root hairs play a key role in the endophytic colonization of olive roots by *Pseudomonas* spp. with biocontrol activity. Microb. Ecol..

[68] Vacheron J., Desbrosses G., Bouffaud M.L., Touraine B., Moënne-Loccoz Y., Muller D., Legendre L., Wisniewski-Dyé F., Prigent-Combaret C. (2013). Plant growth-promoting rhizobacteria and root system functioning. Front. Plant Sci..

[69] Bishnoi U. (2015). PGPR interaction: An ecofriendly approach promoting the sustainable agriculture system. Adv. Bot. Res..

[70] Reddy P.P. (2014). Potential role of PGPR in agriculture. Plant Growth Promoting Rhizobacteria for Horticultural Crop Protection.

[71] Rahimi S., Talebi M., Baninasab B., Gholami M., Zarei M., Shariatmadari H. (2020). The role of plant growth-promoting rhizobacteria (PGPR) in improving iron acquisition by altering physiological and molecular responses in quince seedlings. Plant Physiol. Biochem..

[72] Kumar A., Maurya B.R., Raghuwanshi R. (2014). Isolation and characterization of PGPR and their effect on growth, yield and nutrient content in wheat (*Triticum aestivum* L.). Biocatal. Agric. Biotechnol..

[73] Etesami H., Adl S.M. (2020). Plant growth-promoting rhizobacteria (PGPR) and their action mechanisms in availability of nutrients to plants. Phyto-Microbiome in Stress Regulation.

[74] Anbi A.A., Mirshekari B., Eivazi A., Yarnia M., Behrouzyar E.K. (2020). PGPRs affected photosynthetic capacity and nutrient uptake in different Salvia species. J. Plant Nutr..

[75] Danish S., Zafar-ul-Hye M. (2019). Co-application of ACC-deaminase producing PGPR and timber-waste biochar improves pigments formation, growth and yield of wheat under drought stress. Sci. Rep..

[76] Wang D., Gao Y., Li M., Sturrock C.J., Gregory A.S., Zhang X. (2020). Change in hydraulic properties of the rhizosphere of maize under different abiotic stresses. Plant Soil.

[77] Saleem M., Law A.D., Sahib M.R., Pervaiz Z.H., Zhang Q. (2018). Impact of root system architecture on rhizosphere and root microbiome. Rhizosphere.

[78] Khan N., Zandi P., Ali S., Mehmood A., Adnan Shahid M., Yang J. (2018). Impact of salicylic acid and PGPR on the drought tolerance and phytoremediation potential of *Helianthus annus*. Front. Microbiol..

[79] Vescio R., Malacrinò A., Bennett A.E., Sorgonà A. (2021). Single and combined abiotic stressors affect maize rhizosphere bacterial microbiota. Rhizosphere.

[80] Yadav A.N. (2017). Agriculturally important microbiomes: Biodiversity and multifarious PGP attributes for amelioration of diverse abiotic stresses in crops for sustainable agriculture. Biomed. J. Sci. Tech. Res..

[81] Qu Q., Zhang Z., Peijnenburg W.J.G.M., Liu W., Lu T., Hu B., Chen J., Chen J., Lin Z., Qian H. (2020). Rhizosphere microbiome assembly and its impact on plant growth. J. Agric. Food Chem..

[82] Pérez-Jaramillo J.E., Mendes R., Raaijmakers J.M. (2016). Impact of plant domestication on rhizosphere microbiome assembly and functions. Plant Mol. Biol..

[83] Vives-Peris V., de Ollas C., Gómez-Cadenas A., Pérez-Clemente R.M. (2020). Root exudates: From plant to rhizosphere and beyond. Plant Cell Rep..

[84] Timmusk S., Abd El-Daim I.A., Copolovici L., Tanilas T., Kännaste A., Behers L., Nevo E., Seisenbaeva G., Stenström E., Niinemets Ü. (2014). Drought-tolerance of wheat improved by rhizosphere bacteria from harsh environments: Enhanced biomass production and reduced emissions of stress volatiles. PLoS ONE.

[85] Ali M.A., Naveed M., Mustafa A., Abbas A. (2017). The good, the bad, and the ugly of rhizosphere microbiome. Probiotics and Plant Health.

[86] Zerrouk I.Z., Benchabane M., Khelifi L., Yokawa K., Ludwig-Müller J., Baluska F. (2016). A Pseudomonas strain isolated from date-palm rhizospheres improves root growth and promotes root formation in maize exposed to salt and aluminum stress. J. Plant Physiol..

[87] Nihorimbere V., Ongena M., Smargiassi M., Thonart P. (2011). Beneficial effect of the rhizosphere microbial community for plant growth and health. Biotechnol. Agron. Société Environ..

[88] Grover M., Ali S.Z., Sandhya V., Rasul A., Venkateswarlu B. (2011). Role of microorganisms in adaptation of agriculture crops to abiotic stresses. World J. Microbiol. Biotechnol..

[89] Zia R., Nawaz M.S., Siddique M.J., Hakim S., Imran A. (2020). Plant survival under drought stress: Implications, adaptive responses, and integrated rhizosphere management strategy for stress mitigation. Microbiol. Res..

[90] Dessaux Y., Grandclément C., Faure D. (2016). Engineering the rhizosphere. Trends Plant Sci..

[91] Sharma S., Chandra D., Sharma A.K. (2021). Rhizosphere Plant–Microbe Interactions under Abiotic Stress. Rhizosphere Biology: Interactions between Microbes and Plants.

[92] Mommer L., Hinsinger P., Prigent-Combaret C., Visser E.J. (2016). Advances in the rhizosphere: Stretching the interface of life. Plant Soil.

[93] Li S., Fu Q., Chen L., Huang W., Yu D. (2011). *Arabidopsis thaliana* WRKY25, WRKY26, and WRKY33 coordinate induction of plant thermotolerance. Planta.

[94] Asseng S., Foster I.A.N., Turner N.C. (2011). The impact of temperature variability on wheat yields. Glob. Chang. Biol..

[95] Boo H.O., Heo B.G., Gorinstein S., Chon S.U. (2011). Positive effects of temperature and growth conditions on enzymatic and antioxidant status in lettuce plants. Plant Sci..

[96] Asati A., Pichhode M., Nikhil K. (2016). Effect of heavy metals on plants: An overview. Int. J. Appl. Innov. Eng. Manag..

[97] Halušková L.U., Valentovičová K., Huttová J., Mistrík I., Tamás L. (2010). Effect of heavy metals on root growth and peroxidase activity in barley root tip. Acta Physiol. Plant..

[98] Pavel V.L., Sobariu D.L., Diaconu M., Stătescu F., Gavrilescu M. (2013). Effects of heavy metals on *Lepidium sativum* germination and growth. Environ. Eng. Manag. J. (EEMJ).

[99] Samardakiewicz S., Woźny A. (2005). Cell division in Lemna minor roots treated with lead. Aquat. Bot..

[100] Prasad M.N.V. (2013). Heavy Metal Stress in Plants: From Biomolecules to Ecosystems.

[101] Rahman Z., Singh V.P. (2019). The relative impact of toxic heavy metals (THMs)(arsenic (As), cadmium (Cd), chromium (Cr)(VI), mercury (Hg), and lead (Pb)) on the total environment: An overview. Environ. Monit. Assess..

[102] Nagajyoti P.C., Lee K.D., Sreekanth T.V.M. (2010). Heavy metals, occurrence and toxicity for plants: A review. Environ. Chem. Lett..

[103] Jaishankar M., Tseten T., Anbalagan N., Mathew B.B., Beeregowda K.N. (2014). Toxicity, mechanism and health effects of some heavy metals. Interdiscip. Toxicol..

[104] Nazir R., Khan M., Masab M., Rehman H.U., Rauf N.U., Shahab S., Ameer N., Sajed M., Ullah M., Rafeeq M. (2015). Accumulation of heavy metals (Ni, Cu, Cd, Cr, Pb, Zn, Fe) in the soil, water and plants and analysis of physico-chemical parameters of soil and water collected from Tanda Dam Kohat. J. Pharm. Sci. Res..

[105] Benáková M., Ahmadi H., Dučaiová Z., Tylová E., Clemens S., Tůma J. (2017). Effects of Cd and Zn on physiological and anatomical properties of hydroponically grown *Brassica napus* plants. Environ. Sci. Pollut. Res..

[106] Castillo-Lorenzo E., Pritchard H.W., Finch-Savage W.E., Seal C.E. (2019). Comparison of seed and seedling functional traits in native Helianthus species and the crop *H. annuus* (sunflower). Plant Biol..

[107] Ruíz-Sánchez M., Armada E., Muñoz Y., de Salamone I.E.G., Aroca R., Ruíz-Lozano J.M., Azcón R. (2011). Azospirillum and arbuscular mycorrhizal colonization enhance rice growth and physiological traits under well-watered and drought conditions. J. Plant Physiol..

[108] Saravanakumar D., Kavino M., Raguchander T., Subbian P., Samiyappan R. (2011). Plant growth promoting bacteria enhance water stress resistance in green gram plants. Acta Physiol. Plant..

[109] El-Meihy R.M. (2016). Evaluation of pgpr as osmoprotective agents for squash (*Cucurbita pepo* L.) growth under drought stress. Middle East J..

[110] Gou W., Tian L., Ruan Z., Zheng P.E.N.G., Chen F.U.C.A.I., Zhang L., Cui Z., Zheng P., Li Z., Gao M. (2015). Accumulation of choline and glycinebetaine and drought stress tolerance induced in maize (*Zea mays*) by three plant growth promoting rhizobacteria (PGPR) strains. Pak. J. Bot..

[111] Lim J.H., Ahn C.H., Jeong H.Y., Kim Y.H., Kim S.D. (2011). Genetic monitoring of multi-functional plant growth promoting rhizobacteria Bacillus subtilis AH18 and Bacillus licheniformis K11 by multiplex and real-time polymerase chain reaction in a pepper farming field. J. Korean Soc. Appl. Biol. Chem..

[112] Gupta S., Pandey S. (2019). ACC deaminase producing bacteria with multifarious plant growth promoting traits alleviates salinity stress in French bean (*Phaseolus vulgaris*) plants. Front. Microbiol..

[113] Tolba S.T., Ibrahim M., Amer E.A., Ahmed D.A. (2019). First insights into salt tolerance improvement of Stevia by plant growth-promoting Streptomyces species. Arch. Microbiol..

[114] Habib S.H., Kausar H., Saud H.M. (2016). Plant growth-promoting rhizobacteria enhance salinity stress tolerance in okra through ROS-scavenging enzymes. BioMed. Res. Int..

[115] Ke T., Guo G., Liu J., Zhang C., Tao Y., Wang P., Xu Y., Chen L. (2021). Improvement of the Cu and Cd phytostabilization efficiency of perennial ryegrass through the inoculation of three metal-resistant PGPR strains. Environ. Pollut..

[116] Awan S.A., Ilyas N., Khan I., Raza M.A., Rehman A.U., Rizwan M., Rastogi A., Tariq R., Brestic M. (2020). Bacillus siamensis Reduces Cadmium Accumulation and Improves Growth and Antioxidant Defense System in Two Wheat (*Triticum aestivum* L.) Varieties. Plants.

[117] Akhtar N., Ilyas N., Yasmin H., Sayyed R.Z., Hasnain Z., A Elsayed E., El Enshasy H.A. (2021). Role of *Bacillus cereus* in Improving the Growth and Phytoextractability of *Brassica nigra* (L.) K. Koch in Chromium Contaminated Soil. Molecules.

[118] Belimov A.A., Safronova V.I., Tsyganov V.E., Borisov A.Y., Kozhemyakov A.P., Stepanok V.V., Martenson A.M., Gianinazzi-Pearson V., Tikhonovich I.A. (2003). Genetic variability in tolerance to cadmium and accumulation of heavy metals in pea (*Pisum sativum* L.). Euphytica.

[119] He X., Xu M., Wei Q., Tang M., Guan L., Lou L., Xu X., Hu Z., Chen Y., Shen Z. (2020). Promotion of growth and phytoextraction of cadmium and lead in Solanum nigrum L. mediated by plant-growth-promoting rhizobacteria. Ecotoxicol. Environ. Saf..

[120] Zafar-ul-Hye M., Tahzeeb-ul-Hassan M., Wahid A., Danish S., Khan M.J., Fahad S., Brtnicky M., Hussain G.S., Battaglia M.L., Datta R. (2021). Compost mixed fruits and vegetable waste biochar with ACC deaminase rhizobacteria can minimize lead stress in mint plants. Sci. Rep..

[121] Ashraf A., Bano A., Ali S.A. (2019). Characterisation of plant growth-promoting rhizobacteria from rhizosphere soil of heat-stressed and unstressed wheat and their use as bio-inoculant. Plant Biol..

[122] Abd El-Daim I.A., Bejai S., Meijer J. (2019). Bacillus velezensis 5113 induced metabolic and molecular reprogramming during abiotic stress tolerance in wheat. Sci. Rep..

[123] Khan M.A., Asaf S., Khan A.L., Jan R., Kang S.M., Kim K.M., Lee I.J. (2020). Extending thermotolerance to tomato seedlings by inoculation with SA1 isolate of *Bacillus cereus* and comparison with exogenous humic acid application. PLoS ONE.

[124] Gururani M.A., Upadhyaya C.P., Baskar V., Venkatesh J., Nookaraju A., Park S.W. (2013). Plant growth-promoting rhizobacteria enhance abiotic stress tolerance in Solanum tuberosum through inducing changes in the expression of ROS-scavenging enzymes and improved photosynthetic performance. J. Plant Growth Regul..

[125] Marulanda A., Azcón R., Chaumont F., Ruiz-Lozano J.M., Aroca R. (2010). Regulation of plasma membrane aquaporins by inoculation with a Bacillus megaterium strain in maize (*Zea mays* L.) plants under unstressed and salt-stressed conditions. Planta.

[126] Khan N., Bano A. (2019). Rhizobacteria and abiotic stress management. Plant Growth Promoting Rhizobacteria for Sustainable Stress Management.

[127] Ghosh P.K., De T.K., Maiti T.K. (2018). Role of ACC Deaminase as a Stress Ameliorating Enzyme of Plant Growth-Promoting Rhizobacteria Useful in Stress Agriculture: A Review. Role of Rhizospheric Microbes in Soil.

[128] Niu X., Song L., Xiao Y., Ge W. (2018). Drought-tolerant plant growth-promoting rhizobacteria associated with foxtail millet in a semi-arid agroecosystem and their potential in alleviating drought stress. Front. Microbiol..

[129] Batool T., Ali S., Seleiman M.F., Naveed N.H., Ali A., Ahmed K., Abid M., Rizwan M., Shahid M.R., Alotaibi M. (2020). Plant growth promoting rhizobacteria alleviates drought stress in potato in response to suppressive oxidative stress and antioxidant enzymes activities. Sci. Rep..

[130] Kumar A., Patel J.S., Meena V.S., Srivastava R. (2019). Recent advances of PGPR based approaches for stress tolerance in plants for sustainable agriculture. Biocatal. Agric. Biotechnol..

[131] Shultana R., Tan Kee Zuan A., Yusop M.R., Mohd Saud H., Ayanda A.F. (2020). Effect of salt-tolerant bacterial inoculations on rice seedlings differing in salt-tolerance under saline soil conditions. Agronomy.

[132] Kechid M., Desbrosses G., Rokhsi W., Varoquaux F., Djekoun A., Touraine B. (2013). The NRT 2.5 and NRT 2.6 genes are involved in growth promotion of Arabidopsis by the plant growth-promoting rhizobacterium (PGPR) strain *Phyllobacterium brassicacearum* STM 196. New Phytol..

[133] Bresson J., Vasseur F., Dauzat M., Labadie M., Varoquaux F., Touraine B., Vile D. (2014). Interact to survive: *Phyllobacterium brassicacearum* improves Arabidopsis tolerance to severe water deficit and growth recovery. PLoS ONE.

[134] Galland M., Gamet L., Varoquaux F., Touraine B., Touraine B., Desbrosses G. (2012). The ethylene pathway contributes to root hair elongation induced by the beneficial bacteria *Phyllobacterium brassicacearum* STM196. Plant Sci..

[135] Islam F., Yasmeen T., Ali Q., Ali S., Arif M.S., Hussain S., Rizvi H. (2014). Influence of Pseudomonas aeruginosa as PGPR on oxidative stress tolerance in wheat under Zn stress. Ecotoxicol. Environ. Saf..

[136] Sultana S., Paul S.C., Parveen S., Alam S., Rahman N., Jannat B., Hoque S., Rahman M.T., Karim M.M. (2019). Isolation and identification of salt-tolerant plant growth-promoting rhizobacteria and its application for rice cultivation under salt stress. Can. J. Microbiol..

[137] Rajput L.U.B.N.A., Imran A., Mubeen F., Hafeez F.Y. (2013). Salt-tolerant PGPR strain Planococcus rifietoensis promotes the growth and yield of wheat (*Triticum aestivum* L.) cultivated in saline soil. Pak. J. Bot..

[138] Damodaran T., Sah V., Rai R.B., Sharma D.K., Mishra V.K., Jha S.K., Kannan R. (2013). Isolation of salt tolerant endophytic and rhizospheric bacteria by natural selection and screening for promising plant growth-promoting rhizobacteria (PGPR) and growth vigour in tomato under sodic environment. Afr. J. Microbiol. Res..

[139] Vimal S.R., Singh J.S. (2019). Salt tolerant PGPR and FYM application in saline soil paddy agriculture sustainability. Clim. Chang. Environ. Sustain..

[140] Nawaz A., Shahbaz M., Asadullah A.I., Marghoob M.U., Imtiaz M., Mubeen F. (2020). Potential of salt tolerant PGPR in growth and yield augmentation of wheat (*Triticum aestivum* L.) under saline conditions. Front. Microbiol..

[141] Bal H.B., Nayak L., Das S., Adhya T.K. (2013). Isolation of ACC deaminase producing PGPR from rice rhizosphere and evaluating their plant growth promoting activity under salt stress. Plant Soil.

[142] Egamberdieva D., Wirth S., Bellingrath-Kimura S.D., Mishra J., Arora N.K. (2019). Salt-tolerant plant growth promoting rhizobacteria for enhancing crop productivity of saline soils. Front. Microbiol..

[143] Silambarasan S., Logeswari P., Cornejo P., Kannan V.R. (2019). Role of plant growth–promoting rhizobacterial consortium in improving the *Vigna radiata* growth and alleviation of aluminum and drought stresses. Environ. Sci. Pollut. Res..

[144] Khan M.A., Asaf S., Khan A.L., Adhikari A., Jan R., Ali S., Imran M., Kim K.M., Lee I.J. (2019). Halotolerant rhizobacterial strains mitigate the adverse effects of NaCl stress in soybean seedlings. BioMed Res. Int..

[145] Zhu X., Song F., Xu H. (2010). Influence of arbuscular mycorrhiza on lipid peroxidation and antioxidant enzyme activity of maize plants under temperature stress. Mycorrhiza.

[146] Li L., Ye Y., Pan L., Zhu Y., Zheng S., Lin Y. (2009). The induction of trehalose and glycerol in Saccharomyces cerevisiae in response to various stresses. Biochem. Biophys. Res. Commun..

[147] Paulucci N.S., Gallarato L.A., Reguera Y.B., Vicario J.C., Cesari A.B., de Lema M.B.G., Dardanelli M.S. (2015). Arachis hypogaea PGPR isolated from Argentine soil modifies its lipids components in response to temperature and salinity. Microbiol. Res..

[148] Kang C.H., So J.S. (2016). Heavy metal and antibiotic resistance of ureolytic bacteria and their immobilization of heavy metals. Ecol. Eng..

[149] Issa A., Esmaeel Q., Sanchez L., Courteaux B., Guise J.F., Gibon Y., Ballias P., Clément C., Jacquard C., Vaillant-Gaveau N. (2018). Impacts of Paraburkholderia phytofirmans strain PsJN on tomato (*Lycopersicon esculentum* L.) under high temperature. Front. Plant Sci..

[150] Rodriguez R.J., Henson J., Van Volkenburgh E., Hoy M., Wright L., Beckwith F., Kim Y.O., Redman R.S. (2008). Stress tolerance in plants via habitat-adapted symbiosis. ISME J..

[151] Ali S.Z., Sandhya V., Grover M., Kishore N., Rao L.V., Venkateswarlu B. (2009). Pseudomonas sp. strain AKM-P6 enhances tolerance of sorghum seedlings to elevated temperatures. Biol. Fertil. Soils.

[152] Ali S.Z., Sandhya V., Grover M., Linga V.R., Bandi V. (2011). Effect of inoculation with a thermotolerant plant growth promoting Pseudomonas putida strain AKMP7 on growth of wheat (*Triticum* spp.) under heat stress. J. Plant Interact..

[153] Chang C.H., Yang S.S. (2009). Thermo-tolerant phosphate-solubilizing microbes for multi-functional biofertilizer preparation. Bioresour. Technol..

[154] Desoky E.S.M., Merwad A.R.M., Semida W.M., Ibrahim S.A., El-Saadony M.T., Rady M.M. (2020). Heavy metals-resistant bacteria (HM-RB): Potential bioremediators of heavy metals-stressed *Spinacia oleracea* plant. Ecotox. Environ. Safety.

[155] Ullah S., Ashraf M., Asghar H.N., Iqbal Z., Ali R. (2019). Review Plant growth promoting rhizobacteria-mediated amelioration of drought in crop plants. Soil Environ..

[156] Ghosh D., Gupta A., Mohapatra S. (2019). A comparative analysis of exopolysaccharide and phytohormone secretions by four drought-tolerant rhizobacterial strains and their impact on osmotic-stress mitigation in *Arabidopsis thaliana*. World J. Microbiol. Biotechnol..

[157] Tiwari S., Muthamilarasan M., Lata C. (2021). Genome-wide identification and expression analysis of Arabidopsis GRAM-domain containing gene family in response to abiotic stresses and PGPR treatment. J. Biotechnol..

[158] Merdy P., Gharbi L.T., Lucas Y. (2009). Pb, Cu and Cr interactions with soil: Sorption experiments and modelling. Colloids Surf. A Physicochem. Eng. Asp..

[159] Kang S.M., Shahzad R., Khan M.A., Hasnain Z., Lee K.E., Park H.S., Kim L.R., Lee I.J. (2021). Ameliorative effect of indole-3-acetic acid-and siderophore-producing *Leclercia adecarboxylata* MO1 on cucumber plants under zinc stress. J. Plant Interact..

[160] Javaherdashti R. (2011). Impact of sulphate-reducing bacteria on the performance of engineering materials. Appl. Microbiol. Biotechnol..

[161] Khanna K., Jamwal V.L., Gandhi S.G., Ohri P., Bhardwaj R. (2019). Metal resistant PGPR lowered Cd uptake and expression of metal transporter genes with improved growth and photosynthetic pigments in *Lycopersicon esculentum* under metal toxicity. Sci. Rep..

[162] Gadd G.M., Bahri-Esfahani J., Li Q., Rhee Y.J., Wei Z., Fomina M., Liang X. (2014). Oxalate production by fungi: Significance in geomycology, biodeterioration and bioremediation. Fungal Biol. Rev..

[163] Khan N., Ali S., Tariq H., Latif S., Yasmin H., Mehmood A., Shahid M.A. (2020). Water Conservation and Plant Survival Strategies of Rhizobacteria under Drought Stress. Agronomy.

[164] Etesami H., Maheshwari D.K. (2018). Use of plant growth promoting rhizobacteria (PGPRs) with multiple plant growth promoting traits in stress agriculture: Action mechanisms and future prospects. Ecotoxicol. Environ. Saf..

[165] Arora N.K., Fatima T., Mishra J., Mishra I., Verma S., Verma R., Verma M., Bhattacharya A., Verma P., Mishra P. (2020). Halo-tolerant plant growth promoting rhizobacteria for improving productivity and remediation of saline soils. J. Adv. Res..

[166] Khan N., Bano A. (2019). Exopolysaccharide producing rhizobacteria and their impact on growth and drought tolerance of wheat grown under rainfed conditions. PLoS ONE.

[167] Kumar K., Amaresan N., Madhuri K. (2017). Alleviation of the adverse effect of salinity stress by inoculation of plant growth promoting rhizobacteria isolated from hot humid tropical climate. Ecol. Eng..

[168] ALKahtani M.D., Fouda A., Attia K.A., Al-Otaibi F., Eid A.M., Ewais E.E.D., Hijri M., St-Arnaud M., Hassan S.E.D., Khan N. (2020). Isolation and characterization of plant growth promoting endophytic bacteria from desert plants and their application as bioinoculants for sustainable agriculture. Agronomy.

[169] Tiwari S., Lata C. (2018). Heavy metal stress, signaling, and tolerance due to plant-associated microbes: An overview. Front. Plant Sci..

[170] He Z.L., Yang X.E. (2007). Role of soil rhizobacteria in phytoremediation of heavy metal contaminated soils. J. Zhejiang Univ. Sci. B.

[171] Moreira H., Pereira S.I., Marques A.P., Rangel A.O., Castro P.M. (2016). Selection of metal resistant plant growth promoting rhizobacteria for the growth and metal accumulation of energy maize in a mine soil—Effect of the inoculum size. Geoderma.

[172] Hartman K., Tringe S.G. (2019). Interactions between plants and soil shaping the root microbiome under abiotic stress. Biochem. J..

[173] Chen Y., Palta J.A., Wu P., Siddique K.H. (2019). Crop root systems and rhizosphere interactions. Plant Soil.

[174] Naylor D., Coleman-Derr D. (2018). Drought stress and root-associated bacterial communities. Front. Plant Sci..

[175] Liang J.G., Tao R.X., Hao Z.N., Wang L., Zhang X. (2011). Induction of resistance in cucumber against seedling damping-off by plant growth-promoting rhizobacteria (PGPR) Bacillus megaterium strain L8. Afr. J. Biotechnol..

[176] Rahmoune B., Morsli A., Khelifi-Slaoui M., Khelifi L., Strueh A., Erban A., Kopka J., Prell J., van Dongen J.T. (2017). Isolation and characterization of three new PGPR and their effects on the growth of Arabidopsis and Datura plants. J. Plant Interact..

[177] Turan M., Gulluce M., Cakmakci R., Oztas T., Sahin F., Gilkes R.J., Prakongkep N. The effect of PGPR strain on wheat yield and quality parameters. Proceedings of the 19th World Congress of Soil Science: Soil Solutions for a Changing World.

[178] Erturk Y., Ercisli S., Haznedar A., Cakmakci R. (2010). Effects of plant growth promoting rhizobacteria (PGPR) on rooting and root growth of kiwifruit (*Actinidia deliciosa*) stem cuttings. Biol. Res..

[179] Curá J.A., Franz D.R., Filosofía J.E., Balestrasse K.B., Burgueño L.E. (2017). Inoculation with *Azospirillum* sp.; *Herbaspirillum* sp. bacteria increases the tolerance of maize to drought stress. Microorganisms.

[180] Almaghrabi O.A., Massoud S.I., Abdelmoneim T.S. (2013). Influence of inoculation with plant growth promoting rhizobacteria (PGPR) on tomato plant growth and nematode reproduction under greenhouse conditions. Saudi J. Biol. Sci..

[181] Jones P., Garcia B.J., Furches A., Tuskan G.A., Jacobson D. (2019). Plant host-associated mechanisms for microbial selection. Front. Plant Sci..

[182] De-la-Peña C., Loyola-Vargas V.M. (2014). Biotic interactions in the rhizosphere: A diverse cooperative enterprise for plant productivity. Plant Physiol..

[183] De la Fuente Canto C., Simonin M., King E., Moulin L., Bennett M.J., Castrillo G., Laplaze L. (2020). An extended root phenotype: The rhizosphere, its formation and impacts on plant fitness. Plant J..

[184] Jochum M.D., McWilliams K.L., Borrego E.J., Kolomiets M.V., Niu G., Pierson E.A., Jo Y.K. (2019). Bioprospecting plant growth-promoting rhizobacteria that mitigate drought stress in grasses. Front. Microbiol..

[185] Mishra J., Fatima T., Arora N.K. (2018). Role of secondary metabolites from plant growth-promoting rhizobacteria in combating salinity stress. Plant Microbiome: Stress Response.

[186] Gamez R., Cardinale M., Montes M., Ramirez S., Schnell S., Rodriguez F. (2019). Screening, plant growth promotion and root colonization pattern of two rhizobacteria (*Pseudomonas fluorescens* Ps006 and *Bacillus amyloliquefaciens* Bs006) on banana cv. Williams (*Musa acuminata* Colla). Microbiol. Res..

[187] Kousar B., Bano A., Khan N. (2020). PGPR modulation of secondary metabolites in tomato infested with *Spodoptera litura*. Agronomy.

[188] Vílchez J.I., Yang Y., He D., Zi H., Peng L., Lv S., Kaushal R., Wang W., Huang W., Liu R. (2020). DNA demethylases are required for myo-inositol-mediated mutualism between plants and beneficial rhizobacteria. Nat. Plants.

[189] Zhou D., Huang X.F., Chaparro J.M., Badri D.V., Manter D.K., Vivanco J.M., Guo J. (2016). Root and bacterial secretions regulate the interaction between plants and PGPR leading to distinct plant growth promotion effects. Plant Soil.

[190] Vurukonda S.S.K.P., Vardharajula S., Shrivastava M., SkZ A. (2016). Enhancement of drought stress tolerance in crops by plant growth promoting rhizobacteria. Microbiol. Res..

[191] Naseem H., Ahsan M., Shahid M.A., Khan N. (2018). Exopolysaccharides producing rhizobacteria and their role in plant growth and drought tolerance. J. Basic Microbiol..

[192] Singh B.N., Hidangmayum A., Singh A., Shera S.S., Dwivedi P. (2019). Secondary Metabolites of Plant Growth Promoting Rhizomicroorganisms.

[193] Bakka K., Challabathula D. (2020). Amelioration of Salt Stress Tolerance in Plants by Plant Growth-Promoting Rhizobacteria: Insights from “Omics” Approaches. Plant Microbe Symbiosis.

[194] Lim J.H., Kim S.D. (2013). Induction of drought stress resistance by multi-functional PGPR Bacillus licheniformis K11 in pepper. Plant Pathol. J..

[195] Abbas R., Rasul S., Aslam K., Baber M., Shahid M., Mubeen F., Naqqash T. (2019). Halotolerant PGPR: A hope for cultivation of saline soils. J. King Saud Univ. Sci..

[196] Upadhyay S.K., Singh D.P. (2015). Effect of salt-tolerant plant growth-promoting rhizobacteria on wheat plants and soil health in a saline environment. Plant Biol..

[197] Kumar A., Verma J.P. (2018). Does plant—Microbe interaction confer stress tolerance in plants: A review?. Microbiol. Res..

[198] Li H., Qiu Y., Yao T., Ma Y., Zhang H., Yang X. (2020). Effects of PGPR microbial inoculants on the growth and soil properties of Avena sativa, Medicago sativa, and *Cucumis sativus* seedlings. Soil Tillage Res..

[199] Khan M.N.N., Ahmad Z., Ghafoor A. (2011). Genetic diversity and disease response of rust in bread wheat collected from Waziristan Agency, Pakistan. Int. J. Biodivers. Conserv..

[200] Dimkpa C., Weinand T., Asch F. (2009). Plant–rhizobacteria interactions alleviate abiotic stress conditions. Plant Cell Environ..

[201] Pare P.W., Farag M.A., Krishnamachari V., Zhang H., Ryu C.M., Kloepper J.W. (2005). Elicitors and priming agents initiate plant defense responses. Photosynth. Res..

[202] Yu P., Hochholdinger F. (2018). The role of host genetic signatures on root–microbe interactions in the rhizosphere and endosphere. Front. Plant Sci..

[203] Barea J.M., Pozo M.J., Azcon R., Azcon-Aguilar C. (2005). Microbial co-operation in the rhizosphere. J. Exp. Bot..

[204] Nanjundappa A., Bagyaraj D.J., Saxena A.K., Kumar M., Chakdar H. (2019). Interaction between arbuscular mycorrhizal fungi and *Bacillus* spp. in soil enhancing growth of crop plants. Fungal Biol. Biotechnol..

[205] Ivanov V.B., Bystrova E.I., Seregin I.V. (2003). Comparative impacts of heavy metals on root growth as related to their specificity and selectivity. Russ. J. Plant Physiol..

[206] Sandhya V.S.K.Z., Ali S.Z., Grover M., Reddy G., Venkateswarlu B. (2010). Effect of plant growth promoting *Pseudomonas* spp. on compatible solutes, antioxidant status and plant growth of maize under drought stress. Plant Growth Regul..

[207] Misra J., Pandey V., Singh N. (1994). Effects of some heavy metals on root growth of germinating seeds of *Vicia faba*. J. Environ. Sci. Health Part A.

[208] Luo H., Xu H., Chu C., He F., Fang S. (2020). High temperature can change root system architecture and intensify root interactions of plant seedlings. Front. Plant Sci..

[209] Doty S.L., Oakley B., Xin G., Kang J.W., Singleton G., Khan Z., Vajzovic A., Staley J.T. (2009). Diazotrophic endophytes of native black cottonwood and willow. Symbiosis.

[210] Santos F., Peñaflor M.F.G., Paré P.W., Sanches P.A., Kamiya A.C., Tonelli M., Nardi C., Bento J.M.S. (2014). A novel interaction between plant-beneficial rhizobacteria and roots: Colonization induces corn resistance against the root herbivore *Diabrotica speciosa*. PLoS ONE.

[211] Desbrosses G., Contesto C., Varoquaux F., Galland M., Touraine B. (2009). PGPR-Arabidopsis interactions is a useful system to study signaling pathways involved in plant developmental control. Plant Signal. Behav..

[212] Hassan M.K., McInroy J.A., Kloepper J.W. (2019). The interactions of rhizodeposits with plant growth-promoting rhizobacteria in the rhizosphere: A review. Agriculture.

[213] Rosier A., Medeiros F.H., Bais H.P. (2018). Defining plant growth promoting rhizobacteria molecular and biochemical networks in beneficial plant-microbe interactions. Plant Soil.

[214] Paredes-Páliz K., Rodríguez-Vázquez R., Duarte B., Caviedes M.A., Mateos-Naranjo E., Redondo-Gómez S., Caçador M.I., Rodríguez-Llorente I.D., Pajuelo E. (2018). Investigating the mechanisms underlying phytoprotection by plant growth-promoting rhizobacteria in *Spartina densiflora* under metal stress. Plant Biol..

[215] Mhlongo M.I., Piater L.A., Madala N.E., Labuschagne N., Dubery I.A. (2018). The chemistry of plant–microbe interactions in the rhizosphere and the potential for metabolomics to reveal signaling related to defense priming and induced systemic resistance. Front. Plant Sci..

[216] Igiehon N.O., Babalola O.O. (2018). Below-ground-above-ground plant-microbial interactions: Focusing on soybean, rhizobacteria and mycorrhizal fungi. Open Microbiol. J..

[217] Parmar N., Dufresne J. (2011). Beneficial interactions of plant growth promoting rhizosphere microorganisms. Bioaugmentation, Biostimulation and Biocontrol.

[218] Castro-Sowinski S., Herschkovitz Y., Okon Y., Jurkevitch E. (2007). Effects of inoculation with plant growth-promoting rhizobacteria on resident rhizosphere microorganisms. FEMS Microbiol. Lett..

[219] Liu F.C., Xing S.J., Ma H.L., Du Z.Y., Ma B.Y. (2014). Effects of inoculating plant growth-promoting rhizobacteria on the biological characteristics of walnut (*Juglans regia*) rhizosphere soil under drought condition. Ying Yong Sheng Tai Xue Bao J. Appl. Ecol..

[220] Majeed A., Abbasi M.K., Hameed S., Imran A., Rahim N. (2015). Isolation and characterization of plant growth-promoting rhizobacteria from wheat rhizosphere and their effect on plant growth promotion. Front. Microbiol..

[221] Singh S., Parihar P., Singh R., Singh V.P., Prasad S.M. (2016). Heavy metal tolerance in plants: Role of transcriptomics, proteomics, metabolomics, and ionomics. Front. Plant Sci..

[222] Yadav S.K. (2010). Heavy metals toxicity in plants: An overview on the role of glutathione and phytochelatins in heavy metal stress tolerance of plants. South Afr. J. Bot..

[223] Fahr M., Laplaze L., Bendaou N., Hocher V., El Mzibri M., Bogusz D., Smouni A. (2013). Effect of lead on root growth. Front. Plant Sci..

[224] Chibuike G.U., Obiora S.C. (2014). Heavy metal polluted soils: Effect on plants and bioremediation methods. Appl. Environ. Soil Sci..

[225] Ahmed S., Choudhury A.R., Chatterjee P., Samaddar S., Kim K., Jeon S., Sa T. (2019). The role of plant growth-promoting rhizobacteria to modulate proline biosynthesis in plants for salt stress alleviation. Plant Growth Promoting Rhizobacteria for Sustainable Stress Management.

